# Prospecting Biotechnologically-Relevant Monooxygenases from Cold Sediment Metagenomes: An In Silico Approach

**DOI:** 10.3390/md15040114

**Published:** 2017-04-09

**Authors:** Matías A. Musumeci, Mariana Lozada, Daniela V. Rial, Walter P. Mac Cormack, Janet K. Jansson, Sara Sjöling, JoLynn Carroll, Hebe M. Dionisi

**Affiliations:** 1 Laboratorio de Microbiología Ambiental, Centro para el Estudio de Sistemas Marinos, CONICET, Puerto Madryn, Chubut U9120ACD, Argentina; musumeci@cenpat-conicet.gob.ar (M.A.M.); lozada@cenpat-conicet.gob.ar (M.L.); 2 Área Biología Molecular, Departamento de Ciencias Biológicas, Facultad de Ciencias Bioquímicas y Farmacéuticas, Universidad Nacional de Rosario, CONICET, Suipacha 531 S2002LRK Rosario, Argentina; drial@fbioyf.unr.edu.ar or rial@inv.rosario-conicet.gov.ar; 3 Instituto Antártico Argentino, Ciudad Autónoma de Buenos Aires C1010AAZ, Argentina; wmac@ffyb.uba.ar; 4 Instituto de Nanobiotecnología (NANOBIOTEC), CONICET—Universidad de Buenos Aires, Ciudad Autónoma de Buenos Aires C1113AAD, Argentina; 5 Earth and Biological Sciences Directorate, Pacific Northwest National Laboratory, Richland, WA 99352, USA; janet.jansson@pnnl.gov; 6 School of Natural Sciences and Environmental Studies, Södertörn University, 141 89 Huddinge, Sweden; sara.sjoling@sh.se; 7 Akvaplan-niva, Fram—High North Research Centre for Climate and the Environment, NO-9296 Tromsø, Norway; jolynn.carroll@akvaplan.niva.no; 8 ARCEx—Research Centre for Arctic Petroleum Exploration, Department of Geosciences, UiT The Arctic University of Norway, N-9037 Tromsø, Norway

**Keywords:** bacterial cytochrome P450, Baeyer–Villiger monooxygenases, bioprospecting biocatalysts, phylogenetic analysis, molecular modeling

## Abstract

The goal of this work was to identify sequences encoding monooxygenase biocatalysts with novel features by in silico mining an assembled metagenomic dataset of polar and subpolar marine sediments. The targeted enzyme sequences were Baeyer–Villiger and bacterial cytochrome P450 monooxygenases (CYP153). These enzymes have wide-ranging applications, from the synthesis of steroids, antibiotics, mycotoxins and pheromones to the synthesis of monomers for polymerization and anticancer precursors, due to their extraordinary enantio-, regio-, and chemo- selectivity that are valuable features for organic synthesis. Phylogenetic analyses were used to select the most divergent sequences affiliated to these enzyme families among the 264 putative monooxygenases recovered from the ~14 million protein-coding sequences in the assembled metagenome dataset. Three-dimensional structure modeling and docking analysis suggested features useful in biotechnological applications in five metagenomic sequences, such as wide substrate range, novel substrate specificity or regioselectivity. Further analysis revealed structural features associated with psychrophilic enzymes, such as broader substrate accessibility, larger catalytic pockets or low domain interactions, suggesting that they could be applied in biooxidations at room or low temperatures, saving costs inherent to energy consumption. This work allowed the identification of putative enzyme candidates with promising features from metagenomes, providing a suitable starting point for further developments.

## 1. Introduction

The biotechnological potential of marine bacteria has been exploited in several patented technological processes based on marine enzymes [1]. Metagenomic approaches in poorly characterized marine and coastal environments constitute a promising strategy for the discovery of novel biocatalysts [2]. However, bioprospecting efforts can be hindered by low-coverage sequence information and inefficient read assembly of shotgun sequenced metagenomes as a result of the high diversity of microbial communities in these environments, in particular in sediments [3]. These datasets are comprised mostly of unassembled reads and short scaffolds containing partial protein coding sequences (PCS), with limited biotechnological value. The identification of biocatalyst sequences can also be limited by the low coverage of many metagenomic datasets, as several attractive target enzymes are often encoded in low-abundance members of these highly diverse communities [4]. These methodological restrictions limit the exploitation of the remarkable amount of biotechnologically relevant genetic resources from yet-to-be cultured microorganisms that are currently stored in public metagenomic databases. Furthermore, once a set of sequences is retrieved from the dataset, a knowledge-based selection is required before the synthesis, heterologous expression and characterization of the biocatalyst candidates, in order to avoid the screening of a large number of enzymes, which is expensive and time-consuming [5]. The increasing availability of metagenomic data, coupled to improvements in the design and prediction of protein structures will certainly contribute to improving the initialization steps of directed evolution of protein biocatalysts [6]. 

Oxygenases, which catalyze the addition of oxygen atoms into many organic compounds, show a remarkable enantio- and regio-selectivity and broad substrate specificity [7,8,9,10]. These features make them valuable biocatalysts for the production of synthons relevant for pharmaceutical and chemical industries [4], and it has been suggested that the use of these enzymes will be as prominent as the well-established hydrolases and dehydrogenases [7]. Furthermore, their application in chemical synthesis can replace the use of potentially harmful chemicals (i.e., green chemistry) [8]. Among the most promising group of oxygenases are the Baeyer–Villiger Monooxygenases (BVMOs), well known for their biotechnological potential in the development of pharmaceuticals [11], as well as bacterial cytochrome P450 Monooxygenases (CYP153) [12], which are novel P450 and unique enzymes of the family being soluble and able to hydroxylate highly hydrophobic alkanes [13].

Type I Baeyer–Villiger Monooxygenases (BVMOs) belong to the Group B Flavin-dependent Monooxygenases (B-FDM), a diverse family of enzymes that also includes Flavoprotein Monooxygenases (FMO), N-hydroxylating Monooxygenases (NHMO) and YUCCA Monooxygenases (currently defined as indole 3-pyruvate monooxygenase, EC 1.14.13.168) [14]. BVMOs catalyze the enantio-selective oxidation of ketones to produce esters or lactones, and their applications in organic synthesis have significantly expanded over the last two decades, currently reaching the multi-kilogram scale, and further scale-ups and industrial applications may be expected in the near future [7]. BVMOs have been applied in the production of chiral intermediates for the synthesis of natural products and analogs [15,16,17], the production of Esomeprazole by Codexis being one remarkable example of BVMOs in the industry [18]. With the current trend to evolve the traditional chemical operations toward environmentally harmless processes, the enzymatic Baeyer–Villiger oxidation is promising for a sustainable development of the chemical industry [19].

CYP153 are soluble haem-containing monooxygenases, which catalyze the hydroxylation of medium-chain-length alkanes (from C6 to C11) at the terminal position to produce 1-alkanols. Applications of CYP153 enzymes include the conversion of terpene limonene into perillyl alcohol, a putative anticancer agent as well as the capability to hydroxylate piperidines, pyrrolidines and azetidines to useful pharmaceutical intermediates [13]. Moreover, in the last few years, high sequence diversity was uncovered in genomes and metagenomes for this enzyme group, highlighting their still unexplored potential [20].

The goal of this work was to identify Baeyer–Villiger and CYP153 Monooxygenases in a metagenomic dataset obtained from shotgun metagenomic sequencing of polar and subpolar coastal sediments [21], and to select sequences presenting promising biotechnological features such as wide substrate range, regio-selectivity or novel specificities. Starting from a set of more than 200 putative monooxygenases and using a multi-step approach including phylogenetic analysis and molecular modeling, we selected a total of five sequences, four BVMOs and one CYP153, with evidence of broad or novel substrate specificity. This knowledge-based approach is applicable to other biocatalysts and environments and can provide a systematic framework for bioprospecting efforts in complex metagenomic datasets.

## 2. Results and Discussion

### 2.1. Identification of Metagenomic Sequences

The dataset used in this work for the identification of sequences homologous to monooxygenase enzymes with biotechnological potential contains 23 assembled metagenomes of coastal sediments from four distant polar or subpolar environments (Advent fjord in Spitsbergen, Värtahamnen in the Baltic Sea; Ushuaia Bay in Tierra del Fuego Island and Potter Cove of 25 de Mayo Island). Both marine and brackish ecosystems exposed to oil-pollutants and cold temperatures are represented in this dataset [21]. Microorganisms adapted to extreme conditions are known to produce enzymes with promising features for biotechnological applications [22].

The dataset was queried for sequences encoding putative BVMOs and CYP153 enzymes using Pfam domains and BLAST (Basic Local Alignment Search Tool) searches (Table S1). The number of identified sequences per metagenome varied widely (Table 1). A positive correlation was detected between the total number of oxygenase sequences identified in the metagenomes and the number of PCSs in the assembled metagenomes (Pearson correlation *r* = 0.667, *p* = 0.01). This result suggests that the number of retrieved sequences could have been limited by the relatively low sequence depth (one lane of Illumina HiSeq 1500 per sample), which probably affected the assembly efficiency (Table 1). This is a common problem in metagenomes of samples with high diversity [23]. In spite of this limitation, 264 monooxygenase sequences were identified in the dataset.

### 2.2. BVMOs

#### 2.2.1. Structural Modeling

Considering that some members of B-FDM different from BVMO share the same Pfam domain (PF00743) or have a high sequence homology against BVMOs sequences, the identified metagenomic sequences were further classified by phylogenetic analysis using reference sequences belonging to the different subgroups of B-FDM (Figure S1). The most abundant subgroup represented in this set of metagenomic sequences was BVMO, with 36 PCSs unequivocally assigned to different well-supported clusters containing BVMO references (Figure S1). A cluster of 29 sequences grouped with FMO reference sequences, and 40 metagenomic sequences could not be reliably assigned to any subgroup.

With the aim of identifying novel features related to active sites or ligand specificities, a subset of divergent putative BVMO sequences was chosen for further analysis, based on the following selection criteria. 

**Figure 1:**
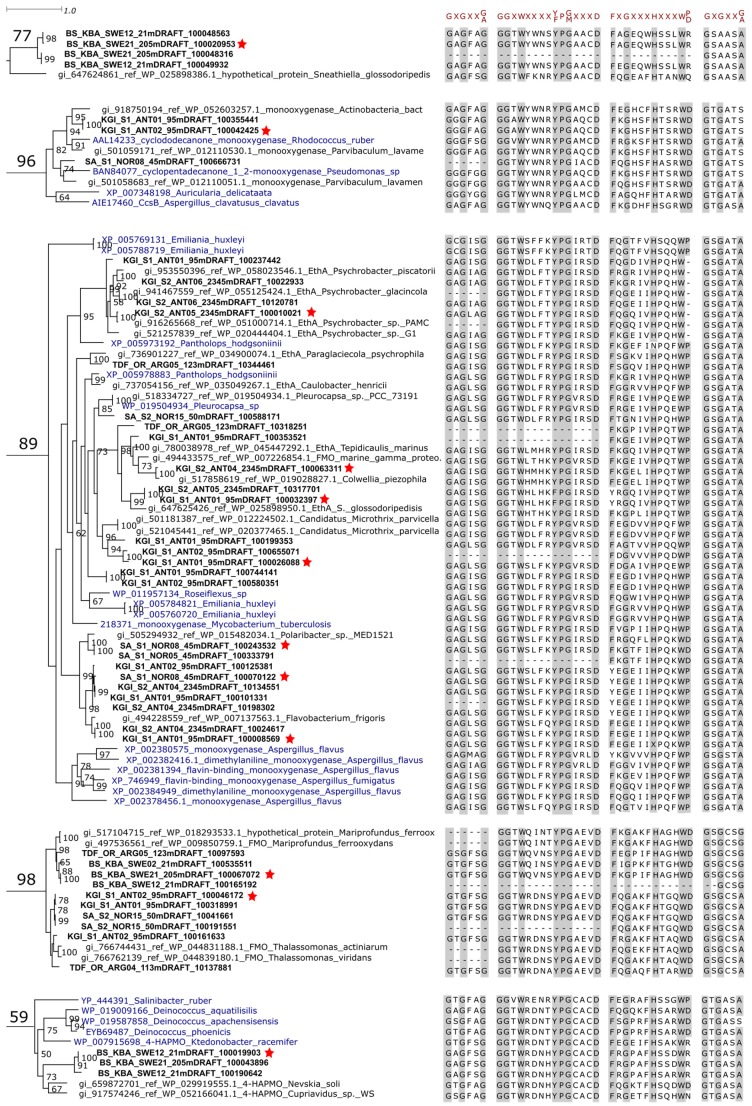
Phylogenetic relationships between metagenomic sequences and BVMO (Baeyer-Villiger Monooxygenase) reference sequences in selected clusters. Clusters were chosen based on the presence of sequences of interest (the full maximum-likelihood phylogenetic tree is shown in Figure S1). Metagenomic sequences and reference BVMO sequences [14,24] are shown in bold and blue, respectively. First matches of metagenomic sequences in BLAST (Basic Local Alignment Search Tool) searches against NCBI (National Center for Biotechnology Information) Genomes database are also shown (in regular font, starting with “the gi” identification number). The stars highlight the putative BVMO sequences selected for further analysis. Numbers in the nodes correspond to bootstrap values (100 replications, only values higher than 50 are shown).

**Figure 2:**
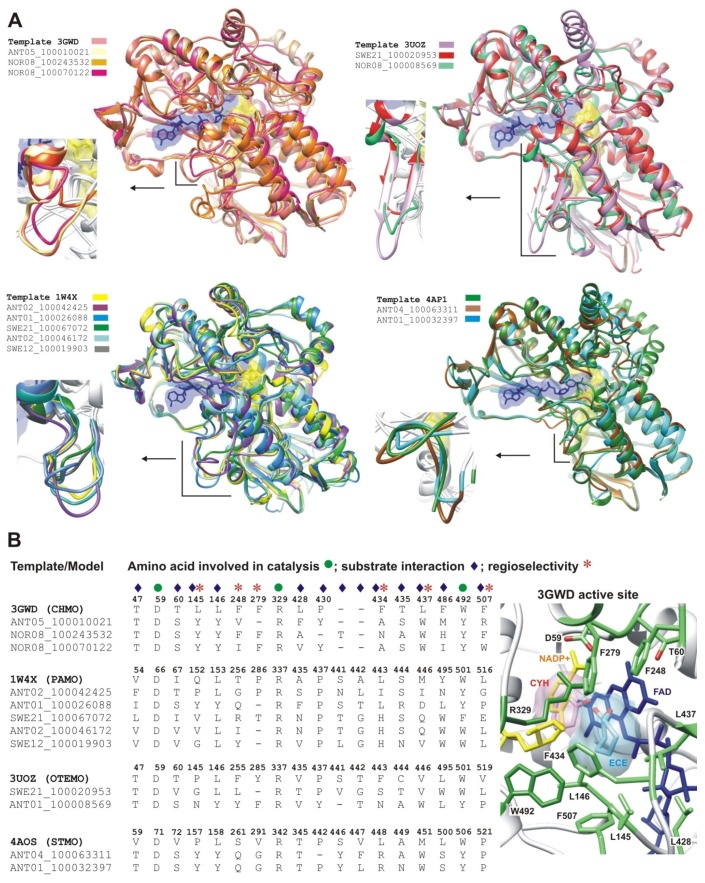
Structural analysis of modeled putative BVMOs. (**A**) structural superimposition between structures that were used as templates for homology modeling and modeled putative BVMOs. FAD (Flavin Adenine Dinucleotide) and NADP^+^ (Nicotinamide Adenine Dinucleotide Phosphate, oxidized form) as found in template structures are shown in yellow and blue, respectively. Comparisons between Control Loops are highlighted in the insets; (**B**) three-dimensional protein structure analysis indicating amino acid residues involved in catalysis, substrate interaction or regioselectivity. The analysis was derived from structural superimposition in (A), comparing amino acid residues of each template structure with structural models. The numbering corresponds to template structures. The active site of CHMO (Cyclohexanone Monooxygenase) from *Rhodococcus* sp. HI-31 [32] is shown as a reference to indicate the spatial arrangement of key amino acids (green). CYH: substrate cyclohexanone (pink). ECE: product ε-caprolactone (cyan). 3UOZ: OTEMO (2-Oxo-∆^3^-4,5,5-trimethylcyclopentenylacetyl-Coenzyme A Monooxygenase) from *Pseudomonas putida* ATCC 17453 [33], 1W4X: PAMO (Phenylacetone Monooxygenase) from *Thermobifida fusca* [34], 4AOS: STMO (Steroid Monooxygenase) from *Rhodococcus rhodochrous* [30].

First, clusters supported by bootstrap values higher than 50% and containing at least one full-length metagenomic sequence were identified in the phylogenetic tree (clusters 77, 96, 89, 98 and 59, Figure S1 and Figure 1). Second, the presence of conserved domains consisting of two Rossmann motifs (GxGxx[G/A]) flanking two BVMO fingerprints ([A/G]GxWxxxx[F/Y]P[G/M]xxxD and FxGxxxHxxxW[P/D]) [24,25,26] was verified in order to confirm their classification as BVMO (Figure 1). Minor differences were observed in some sequences and could be attributed to the divergence of the sequences, considering that residues essential for catalysis remained conserved [24,25]. Full-length metagenomic sequences were then selected from each cluster for modeling the three-dimensional protein structure in detail (Figure 1). In clusters containing metagenomic sequences sharing high identity values (>90.00%), only one representative sequence was selected for structural modeling. Structural models were calculated by homology modeling. Template structures were selected considering coverage, homology percentage and resolution (Table S2). For each metagenomic sequence, 15 models were constructed based on the alignments of models generated using MODELLER, PROMALS3D and MAFFT, and manual adjustments were performed when necessary in order to attain alignment accuracy. The model with the best quality parameters was selected for each of the twelve metagenomic sequences. These selected models were overlapped with their respective template structures for structural comparison (Figure 2A). The structural models of the putative BVMOs showed overall folds similar to those of template structures, with differences in some of the loops that connect α-helixes or β-strands, especially in the so-called “Control Loop” [27] (Figure 2A). This loop influences the active site environment and plays a critical role in enzyme structure and catalysis, mediating NADPH (Nicotinamide Adenine Dinucleotide Phosphate, hydride reduced form) binding and substrate selection [27].

Amino acid residues identified as relevant for catalysis, substrate interaction or stereo-selectivity in crystallized BVMOs [28,29,30,31] were compared with the corresponding residues in the models of metagenomic putative BVMOs (Figure 2B). The residues relevant for catalysis (D, R and W) were conserved in the analyzed metagenomic structural models. Although tyrosines or phenylalanines were observed in some models instead of a conserved catalytic tryptophan involved in NADPH-interaction (W492, 3GWD numbering), the aromatic nature was preserved. However, this difference could influence the co-factor binding mode. More significant differences were observed in residues involved in substrate interaction or regioselectivity (Figure 2A). 

These results suggest that the putative BVMOs identified in the metagenomic dataset could present differences in their substrate profile and/or stereo-selectivity, as well as their NADPH binding mode.

#### 2.2.2. Structural Analysis of the Identified BVMOs: Substrate Range

**Figure 3:**
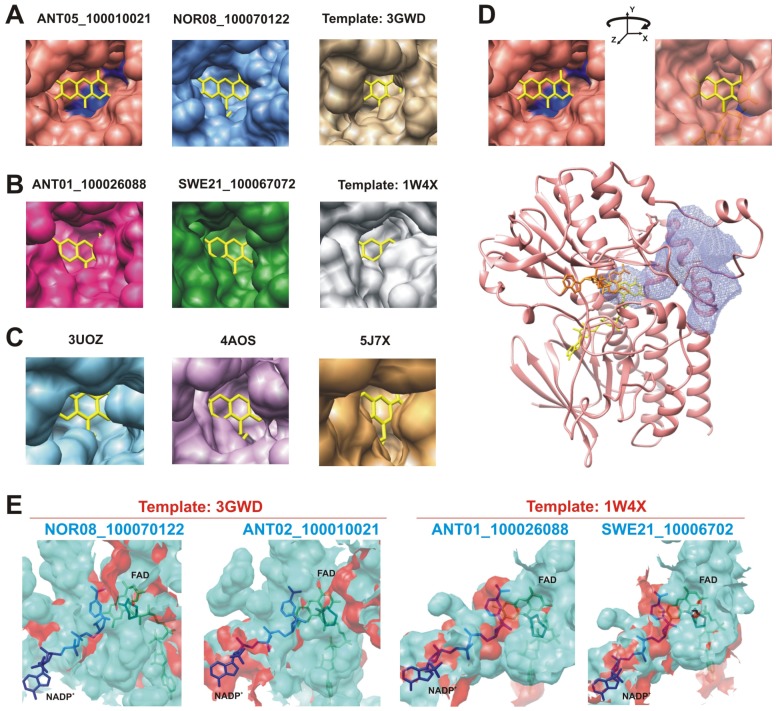
Structural features of four metagenomic putative BVMOs. (**A**) accessibility to the catalytic site of the modeled putative BVMOs ANT05_100010021 and NOR08_100070122 compared with template structure 3GWD; (**B**) comparison of the accessibility to the catalytic site in modeled ANT01_100026088 and SWE21_100067072 with template structure 1W4X; (**C**) accessibility to the catalytic site observed in three additional crystallized BVMOs. 3UOZ: OTEMO from *Pseudomonas putida*; 4AOS: STMO from *Rhodococcus rhodochrous* and 5J7X: BVMO from *Aspergillus flavus*; (**D**) additional site (top, right panel) and protein channel conducting to the catalytic pocket (bottom, light blue surface) identified in the model of ANT05_100010021; (**E**) catalytic pockets of the respective template enzyme (red) compared with the modeled putative BVMOs (cyan). The product ε-caprolactone, such as bound in the crystal structure of CHMO from *Rhodococcus* sp. HI-31 (pdb 4RG3), is shown along with NADP^+^ and FAD. The active sites were identified with CASTp [36] and identified with Chimera [37].

Among the 12 modeled putative metagenomic BVMOs, four (ANT05_100010021, NOR08_100243532, ANT01_10026088 and SWE21_10067072) showed potential structural features different from the template structures (Figure 3). Lesser steric impediments for the accessibility of the substrate to the catalytic FAD prosthetic group were observed in the modeled metagenomic BVMOs compared with their respective templates structures (Figure 3A,B), even including other crystallized BVMOs such as 2-Oxo-∆^3^-4,5,5-trimethylcyclopentenylacetyl-Coenzyme A monooxygenase (OTEMO) and steroid monooxygenase (STMO) (Figure 3C). The 3D model of the putative BVMO ANT05_100010021 showed an additional site for accessing the catalytic FAD, which was identified as a potential protein channel by the software Channel Finder [35] (Figure 3D). In addition, the binding pockets of these putative enzymes were larger than those of the template BVMOs (Figure 3E).

**Figure 4:**
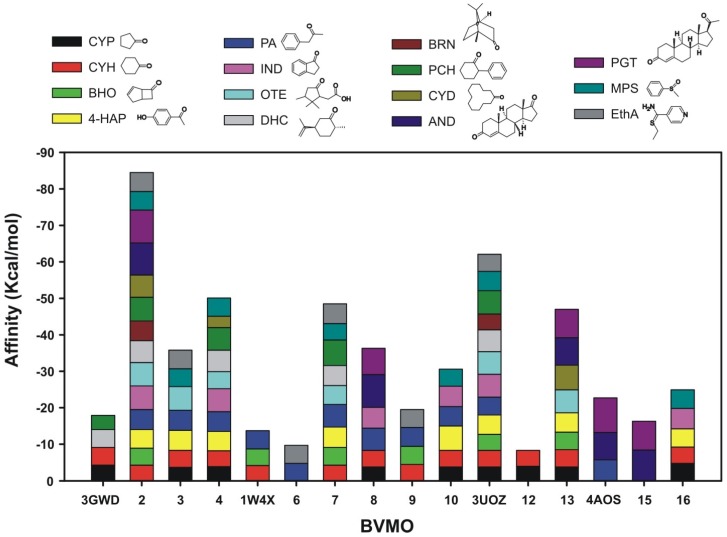
Substrate affinity (kcal/mol) of modeled and crystallized BVMOs estimated by docking analysis. Analyzed substrates are shown at the top. CYP: Cyclopentanone; CYH: Cyclohexanone; BHO: (±)-cisBicyclo[3.2.0]hept-2-en-6-one; 4-HAP: 4-Hydroxyacetophenone; PA: Phenylacetone; IND: Indanone; OTE: 2-Oxo-delta(3)-4,5,5-trimethylcyclopentenylacetic acid; DHC: (1S,4S)-Dihydrocarvone; BRN: Bornanone; PCH: 2-Phenylcyclohexanone; CYD: Cyclododecanone; AND: Androstenedione; PGT: Progesterone; MPS: Methylphenylsulfoxide; Eth: Ethinoamide. 3GWD: CHMO from *Rhodococcus* sp. HI-31 [32]; 2: ANT05_100010021; 3: NOR08_100243532; 4: NOR08_100070122; 1W4X: PAMO from *Thermobifida fusca* [34]; 6: ANT02_100042425; 7: ANT01_100026088; 8: SWE21_100067072; 9: ANT02_100046172; 10: SWE12_100019903; 3UOZ: OTEMO from *Pseudomonas putida* [33]; 12: SWE21_100020953; 13: ANT01_100008569; 4AOS: STMO from *Rhodococcus rhodochrous* [30]; 15: ANT04_100063311; 16: ANT01_100032397. 3D structures of substrate molecules were obtained from PubChem database (compound identification numbers are detailed in Table S3).

The potential capability of the metagenomic BVMOs to bind different substrates was assayed through molecular-docking analyses in order to evaluate whether the potential broader substrate accessibility and larger catalytic pockets observed in the models could influence their substrate range (Figure 4). The orientation of the substrate cyclohexanone, such as those bound in the crystal structure of cyclohexanone monooxygenase (CHMO) from *Rhodococcus* sp. HI-31 (pdb 3UCL), was used as the reference to establish the potentially productive orientations of the ligands for catalysis [31].

**Figure 5:**
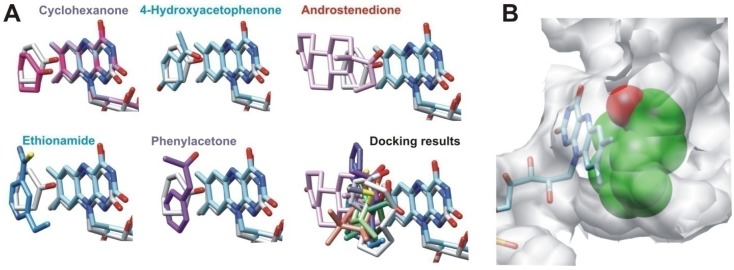
Substrate profile of ANT05_100010021 estimated by molecular-docking analysis. (**A**) obtained spatial arrays, showing results for representative ligands. The results are compared with the geometry adopted by the substrate cyclohexanone in the crystal structure of CHMO from *Rhodococcus* sp. HI-31 (in gray); (**B**) binding of a bulky ligand such as cyclododecanone in the catalytic pocket of the modeled structure.

Docking analysis suggested that three modeled putative BVMOs (ANT05_100010021, NOR08_100070122 and ANT01_100026088) could recognize 14, 10 and nine different substrates of the 15 analyzed, with ANT05_100010021 displaying the broadest substrate range (Figure 4). The docked orientations of the substrates were productive for catalysis (Figure 5A).

The additional site that conducts to the catalytic FAD observed in this putative BVMO could be a route for some substrates to enter the catalytic site, broadening the substrate profile. A similar funnel-shaped cavity leading to the active site, observed in phenylacetone monooxygenase (PAMO) from *Thermobifida fusca* [38], supports this hypothesis. In CHMO and OTEMO, this route is blocked by a dipeptide corresponding to residues 278-279 (3GWD CHMO numbering) that is missing in PAMO [31,38]. In the model of ANT05_100010021, there are no residues in a position homologous to this dipeptide (Figure 2B). In addition, the wide catalytic pocket displayed by the ANT05_100010021 model suggests that it could accommodate larger ligands, such as cyclododecanone, broadening its substrate range (Figure 5B).

#### 2.2.3. Structural Flexibility

The substrate entry path in BVMO enzymes is located at the interface between NADP^+^ and FAD binding domains, where conformational flexibility is high. This flexibility plays an important role in the reshaping of the active site in the presence of different substrates, and has been identified as a structural factor involved in the broad substrate profile displayed by OTEMO BVMOs [33]. Considering the important role of this structural flexibility for catalysis and substrate specificity, the interaction between the FAD and the NADP^+^ binding domains was analyzed. Structural parameters such as putative ΔG^diss^ and ΔG^int^ were calculated (Table 2).

The parameter Δ*G*^diss^ corresponds to the free energy difference between hypothetical dissociated and associated states of FAD and NADP^+^ binding domains. Positive values of Δ*G*^diss^ indicate that an external driving force should be applied in order to dissociate the assembly (i.e., the native enzyme); therefore, assemblies with more positive Δ*G*^diss^ values are thermodynamically more stable. The parameter Δ*G*^int^ indicates the solvation free energy gain upon formation of the assembly and is restricted to the hydrophobic interactions across the interfaces between domains [39]. Both parameters showed lower interaction at the interface in the metagenomic models than in the templates (Table 2). In addition, fewer numbers of hydrogen and salt bridges between FAD and NADP^+^ domains were observed in the modeled putative monooxygenases than in their templates. It has been proposed that the formation and disruption of hydrogen-bonding interactions are relevant for the structural flexibility needed to form the catalytic intermediate in BVMO enzymes [31,34].

The results suggest a higher structural flexibility in the vicinity of the active-site pocket of the metagenomic putative BVMOs (Table 2). This feature could render a broader substrate range, although to the detriment of their structural stability, as indicated by the thermodynamic parameters. The wider substrate accessibility and structural flexibility estimated in the metagenomic putative BVMOs could be a result of adaptation to cold temperatures, as proposed for psychrophilic enzymes [40].

#### 2.2.4. Structural Analysis of Identified BVMOs: Substrate Regioselectivity

The potential regioselectivity of the three putative BVMOs modeled using as template the crystal structure of CHMO from *Rhodococcus* sp. HI-31 bound to the product ε-caprolactone in the tight conformation (pdb 4RG3) (ANT05_100010021, NOR08_100243532 and NOR08_100070122) was assayed by molecular docking analysis. This structure has been proposed to be a suitable scaffold for studying enantio- or regioselectivity [28,41]. The oxidation of (+)-*trans*-dihydrocarvone was used as a model reaction, according to previous studies reported in the literature [28]. This analysis was limited to these putative enzymes because, up to now, the CHMO from *Rhodococcus* sp. HI-31 is the only BVMO for which the structural information necessary for estimating supported product bindings is available. Two regioisomers are possible products of the oxidation of alpha-substituted ketones substrates by a BVMO, the normal lactone formed by insertion of the oxygen atom next to the more substituted carbon and the abnormal lactone formed when the oxygen atom is inserted next to the less substituted carbon. The regioselective oxidation of (+)-*trans*-dihydrocarvone by a set of BVMOs showed that most CHMOs, including the CHMO from *Rhodococcus* sp. HI-31, produce the abnormal lactone only [42]. However, a complete switch of regioselectivity of the CHMO from *Arthrobacter* sp. BP2 toward (+)-*trans*-dihydrocarvone was achieved by site-directed mutagenesis on relevant positions, leading to the normal lactone [28]. The analysis presented in this work suggests that putative BVMOs NOR08_100243532 and NOR_100070122 would be regioselective for abnormal lactones, but NOR08_100243532 would be specific for small molecules (Table 3).

**Figure 6:**
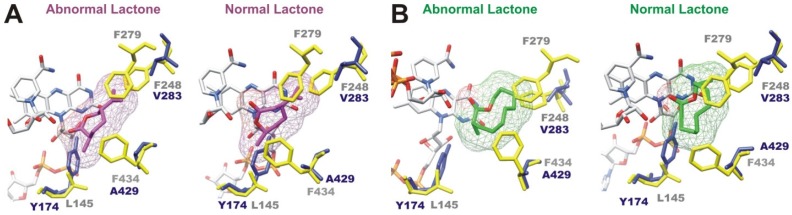
In silico regioselectivity assays of modeled ANT05_100010021 by docking analysis. (**A**) docking results of ANT05_100010021 (blue) with lactone products derived from the oxidation of (+)-*trans*-dihydrocarvone (magenta, mesh representation). Abnormal lactone: (3R,6R)-6-isopropenyl-3-methyloxepan-2-one. Normal lactone: (4R,7R)-4 isopropenyl-7-methyloxepan-2-one; (**B**) docking results of ANT05_100010021 (blue) with lactone products derived from the oxidation of 2-methyl-cyclodecanone (green, mesh representation). Abnormal lactone: 3-methyloxecan-2-one. Normal lactone: 10-methyloxecan-2-one. Only key residues involved in regioselectivity are shown. Structural superimpositions between modeled ANT05_100010021 (blue) and template BVMO (yellow, CHMO from *Rhodococcus* sp. HI-31 in the tight conformation, pdb 4RG3) show differences in amino acids involved in regioselectivity.

These results suggest that ANT05_100010021 would be the only putative BVMO able to produce normal lactone, even from substrates smaller or larger than (+)-*trans*-dihydrocarvone (Table 3). However, this putative enzyme would also be able to produce the abnormal lactones (Table 3), to the detriment of regioselectivity. The presence of gaps or small side-chain amino acids in the modeled ANT05_100010021 instead of bulkier residues typically observed in wild-type CHMOs could prevent steric clashes with lactone products, with the concomitant capability of binding normal lactone (Figure 6). A similar mechanism was proposed for the triple variant of CHMO from *Arthrobacter* sp. BP2 in which the mutation of F248, F279 and F434 to alanine completely switched the regioselectivity of the enzyme and produced the normal lactone [28]. The observed differences in these residues in the structural model of ANT05_100010021 (valine instead of F248, gap instead of F279 and alanine instead of F434) suggest that normal lactone could be produced (Figure 6). However, the resultant wide space in the catalytic cavity could preclude regioselectivity. 

### 2.3. Cytochrome P450 CYP153

**Figure 7:**
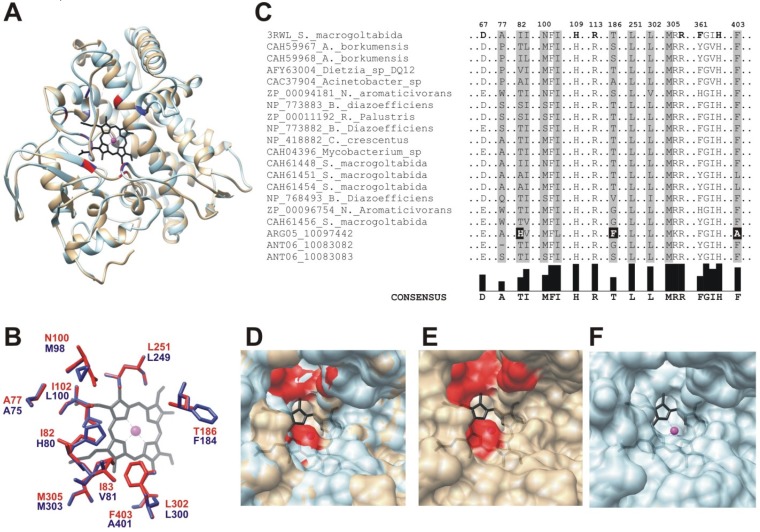
Structural features of a metagenomic putative cytochrome P450 CYP153. (**A**) structural superimposition between the modeled ARG05_10097442 (light blue) and template (grey) CYP153. The haem prosthetic group is shown in black in the center of the structure, with the iron atom as magenta sphere; (**B**) alignment of the characterized CYP153 and the metagenomic sequences. Amino acids involved in haem interaction are in bold. The amino acids relevant for stereo-specificity are in grey boxes, with the number relatives to the crystal structure of CYP153 from *Sphingopyxis macrogoltabida* (pdb 3RWL). As shown in white, the sequence ARG05_10097442 displays unique amino acid modifications; (**C**) spatial arrangement of the amino acids relevant for stereo-specificity (highlighted in grey boxes in Figure 5B) of the template (blue, 3RWL), compared with the model (red); (**D**) surface representation of the overlapped active sites of the templates and model; (**E**) active site of the template (3RWL), showing in red the amino acids relevant for stereo-specificity; (**F**) active site of the modeled ARG05_10097442.

The cytochrome P450 family comprises a wide variety of enzymes including CYP153, which are able to hydroxylate medium-long alkanes (C_5_ to C_16_) at the terminal carbon [43]. Out of the 156 PCS for putative cytochrome P450 identified in the metagenomic dataset, nine clustered with sequences from characterized CYP153 enzymes (Figure S2). Two of these sequences grouped with the phylogenetic cluster II defined by Nie and collaborators [20] and the other seven with the cluster IV. Only the metagenomic sequences ARG05_10097442 (cluster II), ANT06_10083082 and ANT06_10083083 (cluster IV) were full-length, and were modeled using the crystal structure of the cytochrome P450pyr hydroxylase from *Sphingomonas* sp. HXN-200 (pdb 3RWL) [44]. The application of this enzyme for the synthesis of (S)-N-benzyl 3-hydroxy-pyrrolidine has been reported [44], which is a useful intermediate for the preparation of several pharmaceutical products, antibiotic drugs and agricultural chemicals [45]. To improve its performance as a biocatalyst, this enzyme has been engineered by iterative saturation mutagenesis and the critical amino acids for sterero-specificity were identified [44,46]. These residues surround the catalytic haem and are critical not only for sterero-specificity but also for substrate specificity. The 3D model of ARG05_10097442 is shown in Figure 7A, and similar results were obtained with other putative CYP153 enzyme sequences (data not shown).

Different residues were observed in the model with respect to template structure at amino acid residues relevant for stereo-specificity and substrate specificity (Figure 7B). In order to determine if these amino acid differences are present in other CYP153s, the sequences identified in this study were compared with characterized CYP153s [20] (Figure 7C). The sequence ARG05_10097442 showed differences in the positions of the amino acids relevant for stereo-specificity (black boxes, Figure 7C), which were not found by saturation mutagenesis of P450pyr hydroxylase from *Sphingomonas* sp. HXN-200 [44,46]. The residues observed in the sequence ARG05_10097442 could reduce steric impairments around the catalytic haem (Figure 7D–F), allowing changes in catalytic properties related to substrate/product features and probably a novel substrate/product stereo-specificity.

## 3. Materials and Methods

### 3.1. Characteristic of Cold Sediments’ Metagenomic Dataset 

The metagenomic dataset analyzed in this study was obtained by shotgun sequencing of DNA isolated from 23 sediment samples retrieved from four high-latitude coastal environments: (i) Advent Fjord, Spitsbergen, Svalbard Archipelago, Norway [NOR]; (ii) Port Värtahamnen, Stockholm, Baltic Sea, Sweden [SWE]; (iii) Ushuaia Bay, Tierra del Fuego Island, Argentina [ARG]; and (iv) Potter Cove, 25 de Mayo (King George) Island, Antarctica [ANT]. Details on sampling, DNA extraction and shotgun sequencing were previously reported [21]. Briefly, shotgun sequencing was performed using Illumina HiSeq 1500 (2 × 150-bp paired end reads, one lane per sample), at the facilities of the Joint Genome Institute, Department of Energy, 2800 Mitchell Drive, Walnut Creek, CA, USA. The 23 metagenomes, including unassembled reads and scaffolds, were annotated using the IMG (Integrated Microbial Genomes) pipeline [47]. This dataset contains a total of 13,931,912 CDS (coding sequences) in the assembled fraction [21]. The sequences are available at the IMG/M (Integrated Microbial Genomes and Microbiomes) server [47] under accession numbers 3300000118-3300000136, 3300000241-3300000243, and 3300000792.

### 3.2. Screening of the Metagenomic Dataset

For each enzyme family (BVMO and CYP153), sequences were obtained from the metagenomes using two complementary functional evidences [47]. Firstly, sequences containing Pfam domains [48] PF00067 (“Cytochrome P450”) or PF00743 (“Flavin-binding monooxygenase-like”) were selected and downloaded from the IMG server. Secondly, BLASTP searches with a cut-off E-value of 10^−5^ were performed in IMG, using selected sequences from crystallized and/or biochemically characterized enzymes as query (Table S1). Duplicated sequences were eliminated and full-length or nearly full-length sequences were retrieved from the datasets by preselecting sequences longer than 250 or 300 amino acids, depending on the enzyme (Table S1). An additional filtering was applied to metagenomic P450 sequences, as the P450 superfamily included more than 1000 families of bacteria alone [49]. Therefore, a representative set of 1,122 bacterial P450 sequences was downloaded from the bacterial cytochrome P450 database [49], and only sequences whose first match in a standalone blast analysis corresponded to CYP153 family members in the database were kept.

### 3.3. Phylogenetic Analysis

Reference sequences for each enzyme family were collected from the literature. Sequences coding for cytochrome P450 were obtained from a recent work published by Nie et al. [20]. For BVMOs, an in-house-built database was constructed from sequences previously selected by Huijbers et al. and Mascotti et al. [14,24]. In addition, BLASTP first matches of the obtained metagenomic sequences against NCBI (National Center for Biotechnology Information, Bethesda, MD, USA) Representative Genomes database were also added to the analysis, as many of the sequences were found to be divergent from those chosen as reference. Reference sequences were aligned in ClustalX [50] with default parameters, followed by multiple alignment of the identified metagenomic sequences and their first matches against the reference alignment, using ClustalX. Sequence alignments were manually trimmed in order to obtain an equal number of positions, and maximum-likelihood phylogenetic trees were constructed in RAxML version 8.2.3 (Heidelberg Institute for Theoretical Studies, D-69118 Heidelberg, Germany) [51], with GAMMA model of rate heterogeneity, LG substitution matrix and empirical base frequencies (option PROTGAMMALGF). Bootstrapping was performed with 100 replications. 

### 3.4. Three-Dimensional Protein Structure Modeling and Model Quality Evaluation

For each metagenomic sequence, the HHpred web server was used to search for suitable templates for building high quality models [52]. Selected templates presented the highest sequence identity, coverage and resolution (Table S2). For each metagenomic sequence, five models were calculated using MODELLER version 9.16 (Departments of Biopharmaceutical Sciences and Pharmaceutical Chemistry, and California Institute for Quantitative Biomedical Research, Mission Bay Byers Hall, University of California San Francisco, San Francisco, CA, USA) [53]. Models were ranked using DOPE (Discrete Optimized Protein Energy) Z scores. The top models with the lowest energy scores for each metagenomic sequence were further validated by Verify3D [54] and PROCHECK [55]. A final step of refinement was performed with the server 3Drefine [56]. Clashes remotion was carried out using Chimera [37] and Swiss PDB (Protein Data Bank files) viewer [57]. Energy minimization was performed with YASARA (Yet Another Scientific Artificial Reality Application) [58].

### 3.5. Docking Analysis

Three-dimensional structure files of ligand molecules were downloaded from the PubChem database [59], and compound identifier numbers (CID) are detailed in Table S3. Docking analyses were carried out using the software AutoDock Vina (Molecular Graphics Lab at The Scripps Research Institute, La Jolla, CA, USA) with an exhaustiveness of 8.0 [60]. Modeled structures were superimposed to template structures co-crystallized with substrates (or products) in functional orientation. The different ligands to be in silico assayed were manually superimposed to the substrate (or product) bound to the active site. The enzyme and ligand molecules were saved separately as different PDB files and further loaded in AutoDock Vina. Grid parameters were set using coordinates comprising the active site. Only affinity values corresponding to geometry bindings compatible for catalysis were considered.

### 3.6. Calculation of Structural Parameters

Parameters associated with protein structure stability were calculated using PDBePISA server (Protein Interfaces, Surfaces and Assemblies) [39]. For calculations, FAD and NADP^+^ binding subdomains were considered as different interacting protein chains. The information to define subdomains was obtained from the crystal structure of the different Baeyer–Villiger Monooxygenase used as templates [33]. For the modeled enzymes, the same space symmetry groups of the crystal structures used as templates were considered. The catalytic pockets were calculated with the CASTp server using a probe radius of 1.4 Å [36]. The obtained poc files were analyzed with the software chimera [37]. The identification of protein channels was carried out with the software Channel Finder (The Scripps Research Institute and Program in Computational Biology and Bioinformatics New Haven, CT, USA) of the Web server 3V, using 10 and 3 as outer and inner probe radius, respectively [35].

## 4. Conclusions

The methodological framework reported in this work allowed the selection of four sequences out of ~14 million PCSs as candidates for synthesis and heterologous expression, greatly aiding in the efficient and knowledge-based exploitation of a highly fragmented metagenomic dataset. These sequences include the putative BVMOs ANT05_100010021, NOR08_100070122 and ANT01_100026088, which were predicted to have a wide substrate range and novel substrate specificities. In addition, NOR08_100070122 and NOR08_100243532 could be regioselectives for abnormal lactones. On the other hand, the putative CYP153 ARG05_10097442 could present novel substrate specificity and regioselectivity. These putative enzymes could be used in the synthesis of a wide variety of lactones or 1-alkanols at room or low temperatures, where biooxidations may produce better results than the typical chemical oxidation with regard to performance and cost savings in energy consumption. These putative enzymes also could be applied to improve procedures aimed to overcome substrate and product inhibition, performing the reaction in a biphasic system by using organic solvents whose water miscibility can be varied by temperature [19]. Considering that the enzymes could catalyze the reaction at low temperatures, upon this condition, the solubility of the substrate in the aqueous phase would be diminished, improving the feature of the organic phase to trap the substrate during the reaction and favoring the extraction of the product from the aqueous phase.

The presented evidence suggests that the active sites of the selected putative monooxygenases would be larger and more accessible than those from previously biochemically characterized enzymes. It has been observed that low temperature adapted enzymes present larger catalytic cavities, more accessible to ligands, than mesophilic enzymes [40]. A better accessibility is suggested not only to be responsible for the accommodation of the substrate at low energy cost but also to facilitate the release and exit of the reaction products [40]. Structural flexibility also plays a relevant role for cold adaptation because it involves a tuned destabilization of the active site or the whole protein, allowing the catalytic center to be more mobile or flexible at temperatures that tend to freeze molecular motions [40]. In BVMOs, the structural plasticity represents an advantage for catalysis considering the proposed need of these enzymes to bind cofactors and substrates with diverse structures in order to catalyze the elaborated chemical mechanism [31]. These features were observed in the modeled enzymes, suggesting structural mechanisms for cold adaptation. These structural features could favour oxygenation reactions at low or medium temperature with a wide range of substrates. Moreover, novel stereo-specificities could be potentially obtained as well, considering that BVMOs are continuously evolving to acquire new activities, depending on the emerging availabilities of new compounds in the natural environment [30].

Finally, the methodology applied in this work can be used for mining genetic information with biotechnological potential from metagenomic datasets currently stored in public databases, as well as to complement approaches based on functional metagenomics or where only specific HMMs are constructed and applied in screening. This complement may strengthen the prediction aimed to rationale biocatalysts selection before biochemical characterization.

## Figures and Tables

**Table 1:** Number of retrieved monooxygenase sequences in polar and sub-polar coastal sediment metagenomes.

Sample	B-FDM ^a^	CYP153 ^b^	Identified Sequences/Metagenome	PCS ^c^	Assembly (%) ^d^
NOR02	0	0	0	2.82 × 10^5^	12.99
NOR05	8	3	11	7.85 × 10^5^	22.74
NOR08	11	15	26	1.28 × 10^6^	29.04
NOR13	2	1	3	3.13 × 10^5^	17.00
NOR15	9	7	16	1.47 × 10^6^	34.55
NOR18	3	1	4	3.92 × 10^5^	23.55
SWE02	2	5	7	1.11 × 10^6^	19.29
SWE07	0	0	0	2.68 × 10^5^	8.77
SWE12	7	1	8	1.08 × 10^6^	18.17
SWE21	6	3	9	8.66 × 10^5^	17.10
SWE26	0	0	0	5.39 × 10^5^	12.03
ARG01	0	1	1	1.39 × 10^5^	4.50
ARG02	0	1	1	1.74 × 10^5^	5.20
ARG03	0	0	0	4.74 × 10^5^	13.86
ARG04	1	3	4	2.79 × 10^5^	8.15
ARG05	6	8	14	7.13 × 10^5^	13.81
ARG06	0	0	0	1.87 × 10^5^	5.21
ANT01	19	32	51	1.11 × 10^6^	29.19
ANT02	10	23	33	9.72 × 10^5^	27.56
ANT03	1	4	5	2.79 × 10^5^	13.90
ANT04	6	19	25	6.57 × 10^5^	26.45
ANT05	6	12	18	4.67 × 10^5^	20.40
ANT06	11	17	28	1.01 × 10^5^	7.80
Total	108	156	264	1.39 × 10^7^	

^a^ B-FDM: Flavin-Dependent Monooxygenases; ^b^ CYP153: Bacterial Cytochrome P450; ^c^ PCS: Number of protein coding sequences in the assembled metagenomes; ^d^ Assembly (%) as percentage of reads mapping the scaffolds.

**Table 2:** Structural features of modeled BVMOs (Baeyer-Villiger Monooxygenases) and their respective templates.

Enzyme	Δ*G*^int (a)^ (kcal/mol)	Δ*G*^diss (b*)*^ (kcal/mol)	*N*_HB_^(c)^	*N*_SB_^(d)^
3GWD	−30.8	35.2	38	6
NOR08_100243532	−26.5	22.6	21	0
ANT05_100010021	−22.1	17.7	18	5
NOR08_100070122	−28.1	23.5	18	3
1W4X	−51.3	28.3	49	5
ANT01_100026088	−17.7	10.7	13	4
SWE21_100067072	−24.1	18.3	22	7
3UOZ	−30.5	33.2	35	4

**^(a)^** Δ*G*^int^: Solvation free energy gain upon formation of the interface. Negative values correspond to positive protein affinity; **^(b)^** Δ*G*^diss^: Free energy of dissociation. Assemblies with Δ*G*^diss^ > 0 are thermodynamically stable; **^(c)^** *N*_HB_: Number of potential hydrogen bonds across the interface; **^(d)^** *N*_SB_: Number of potential salt bridges across the interface; 3GWD: CHMO from *Rhodococcus* sp. HI-31; 1W4X: PAMO from *Thermobifida fusca*; 3UOZ: OTEMO from *Pseudomonas putida.*

**Table 3:** Affinities of modeled putative BVMOs and CHMO (Cyclohexanone Monooxygenase) from *Rhodococcus* sp. HI-31 by products as estimated by docking analysis (kcal/mol).

Structure of Lactone	BVMO			
Products	ANT05_100010021	NOR08_100243532	NOR08_100070122	4RG3
A- 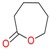	−4.5	-4.7	-5.0	-6.0
B- (Normal) 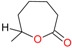	−5.3	NB	NB	NB
C- (Abnormal) 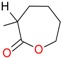	−5.1	-5.0	-5.5	-6.2
D- (Normal) 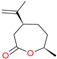	−6.1	NB	NB	NB
E- (Abnormal) 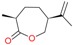	−5.8	NB	-5.9	-4.5
F- (Normal) 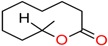	−6.9	NB	NB	NB
G- (Abnormal) 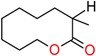	−4.9	NB	-6.2	NB

A: ε-Caprolactone; B: 3-Methyloxepan-2-one; C: 7-Methyl-2-oxepanone; D: (4R,7R)-4 isopropenyl-7-methyloxepan-2-one; E: (3R,6R)-6-isopropenyl-3-methyloxepan-2-one; F: 10-methyloxecan-2-one; G: 3-methyloxecan-2-one. NB: No binding. 4RG3: CHMO from *Rhodococcus* sp. HI-31 bound to ε-caprolactone [41]. 3D structures of product molecules were obtained from PubChem database (compound identification numbers are detailed in Table S3).

## Supplementary Materials

The following are available online at www.mdpi.com/1660-3397/15/4/114/s1. Table S1. Functional evidence and constraints used for retrieving metagenomic sequences. Figure S1. Phylogenetic tree of metagenomic sequences and Group B Flavin-dependent Monooxygenase reference sequences. Metagenomic sequences are shown in bold. Reference sequences for NHMO, FMO and BVMO [14,24] are shown in cyan, green and blue, respectively. Orange corresponds to outgroup sequences (Class A Flavin-dependent Monooxygenases). Sequences identified as first matches in a BLASTP search of metagenomic sequences against NCBI Representative Genomes database are shown starting with the gi identification. Red stars highlight metagenomic sequences modeled in this work and green circles indicate crystallized BVMOs. The phylogenetic tree was constructed by maximum-likelihood in RaXML. Bootstrapping was performed with 100 replications, and only bootstrap values higher than 50 are shown in the nodes. Table S2. Parameters calculated for selection of templates for homology modeling. Only the top two template structures are shown. Figure S2. Phylogenetic tree of metagenomic sequences clustering with cytochrome P450 reference sequences. Metagenomic sequences grouping with CYP153 reference sequences are in bold. Full-length sequences selected for further analysis are identified with red stars. Table S3. Compound identification numbers (CID) in PubChem database of ligands (substrates and products) assayed in docking analysis.
