# Supplementary material for: Prospecting Biotechnologically-Relevant Monooxygenases from Cold Sediment Metagenomes: An In Silico Approach
Source: Mar Drugs. 2017 Apr 9;15(4):114. doi: 10.3390/md15040114 (PMC5408260; doi:10.3390/md15040114)
Supplement: Supplementary file 1 [file marinedrugs-15-00114-s001.pdf]

Table S1. Functional evidences and constraints used for retrieving metagenomic sequences.

	Cluster/subgroup	Pfam	BLAST query	Cut-off length (aa)
**P450**	I	PF00067	Cytochrome P450 from *Mycobacterium* (GB WP_011560601.1)	300
	II	PF00067	Cytochrome P450 from *Parvibaculum lavamentivorans* (GB WP_012110862.1)	300
	III	PF00067	Cytochrome P450 from *Phenylobacterium zucineum* (GB WP_012521375.1)	300
	IV	PF00067	Cytochrome P450 from *Sphingopyxis alaskensis* (WP_011543134.1)	300
**FDM - BVMO**	Cyclohexanone monooxygenase (CHMO)	PF00743	CHMO from *Rhodococcus* sp. HI-31 (GB BAH56677)	250
	Steroid monooxygenase (STMO)	PF00743	Steroid monooxygenase from *Rhodococcus rhodochrous* (GB BAA24454)	250
	Hydroxyacetophenone monooxygenase (HAPMO)	PF00743	HAPMO from *Pseudomonas fluorescens* (GB Q93TJ5.1)	250
	Phenylacetone monooxygenase (PAMO)	PF00743	PAMO from T*hermobifida fusca* (GB Q47PU3)	250

aa: amino acid. FDM-BVMO: Flavin-dependent Monooxygenases, Baeyer-Villiger type. GB: GenBank accession number.

Figure S1. Phylogenetic tree of metagenomic sequences and Group B Flavin-dependent Monooxygenase reference sequences. Metagenomic sequences are shown in bold. Reference sequences for NHMO, FMO and BVMO [1,2] are shown in cyan, green and blue respectively. Orange corresponds to outgroup sequences (Class A Flavin-dependent Monooxygenases). Sequences identified as first matches in a BLASTP search of metagenomic sequences against NCBI Representative Genomes database are shown starting with the gi identification. Red stars highlight metagenomic sequences modeled in this work and green circles indicate crystallized BVMOs. The phylogenetic tree was constructed by maximum-likelihood in RaXML. Bootstrapping was performed with 100 replications, only bootstrap values higher than 50 are shown in the nodes.

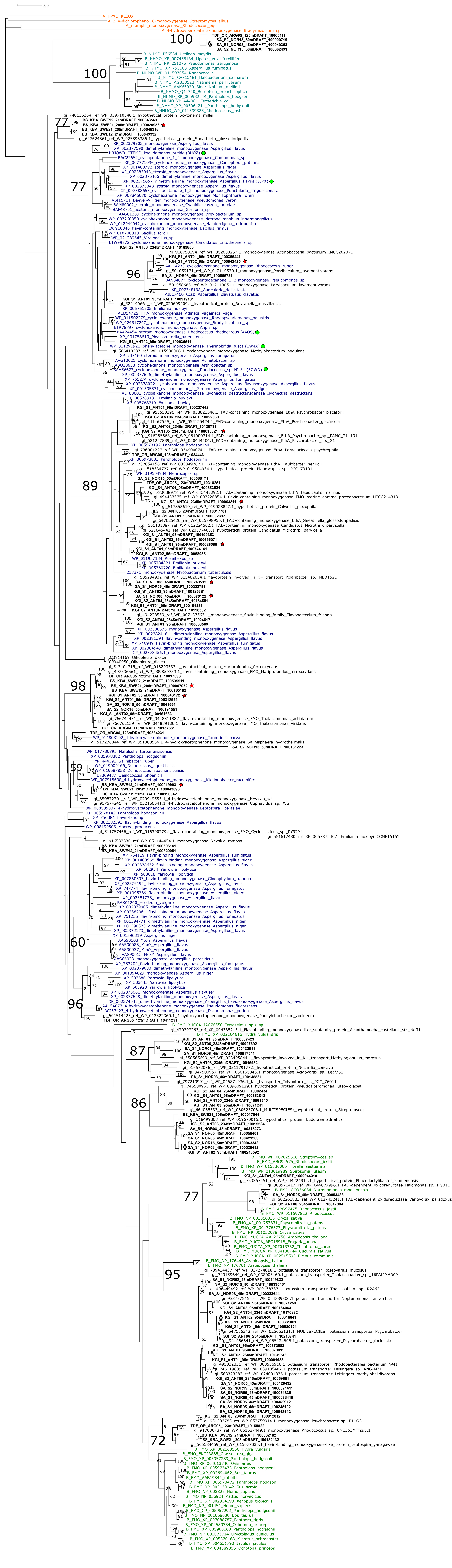

Table S2. Parameters calculated for selection of templates for homology modeling [a]. Only the two top template structures are shown.

Sequence	length (aa)	Top Hits	Score	seq ID %	query HMM	coverage %	template HMM	coverage %
NOR08_100070122	482	**3gwd**	476.8	23	1-475	98.55	12-539 (548)	96.17
		3uox	473.3	24	1-477	98.96	5-545 (545)	99.08
NOR08_100243532	484	1w4x	480,6	23	1-477	98.55	12-542 (542)	97.79
		**3gwd**	485	24	1-478	98.76	8-541 (548)	97.09
ANT01_100008569	483	5j7x	479.1	23	1-478	88.03	8-541 (549)	97.09
		**3uox**	469.9	26	1-479	88.21	5-544 (545)	98.90
ANT01_100032397	527	3uox	480.1	24	36-520	91.84	1-543 (545)	99.45
		**4ap1**	477.0	25	24-520	94.12	1-545 (549)	99.09
ANT04_100063311	499	1w4x	470.2	23	1-492	98.40	1-540 (542)	99.45
		**4ap1**	462.6	24	1-492	98.40	6-545 (549)	98.18
ANT01_100026088	510	**1w4x**	492.1	25	11-498	95.00	2-540 (542)	99.00
		4ap1	485.4	23	7-498	96.00	3-545 (549)	99.00
ANT05_100010021	527	**3gwd**	501.1	20	20-520	95.00	1-529 (541)	98.00
		5j7x	505.6	20	17-522	96.00	2-541 (549)	98.00
SWE12_100019903	489	**1w4x**	603.6	32	1-487	99.00	9-538 (542)	98.00
		4ap1	595.1	32	1-487	99.00	14-543 (549)	96.00
SWE21_100067072	524	**1w4x**	575.9	26	25-523	95.00	9-542 (542)	98.00
		3gwd	574.6	26	21-524	96.00	5-542 (548)	98.00
ANT02_100046172	499	**1w4x**	596.1	27	1-498	99.00	10-542 (542)	98.00
		3gwd	591.3	26	1-499	100.00	10-542 (548)	97.00
SWE21_100020953	492	1w4x	598.2	31	2-491	99.59	8-542 (542)	99.00
		**3uox**	594.7	30	2-491	99.00	1-545 (545)	100.00
ANT02_100042425	609	**1w4x**	606.2	32	58-601	89.16	12-542 (542)	97.78
		5m10	599.4	29	58-607	90.14	4-539 (541)	98.89

[a] Parameters were calculated by using the server HHPred [3].

In bold the templates selected for homology modeling of each metagenomic sequence are highlighted.

Figure S2. Phylogenetic tree of metagenomic sequences clustering with cytochrome P450 reference sequences. Metagenomic sequences grouping with CYP153 reference sequences are in bold. Full-length sequences selected for further analysis are identified with red stars

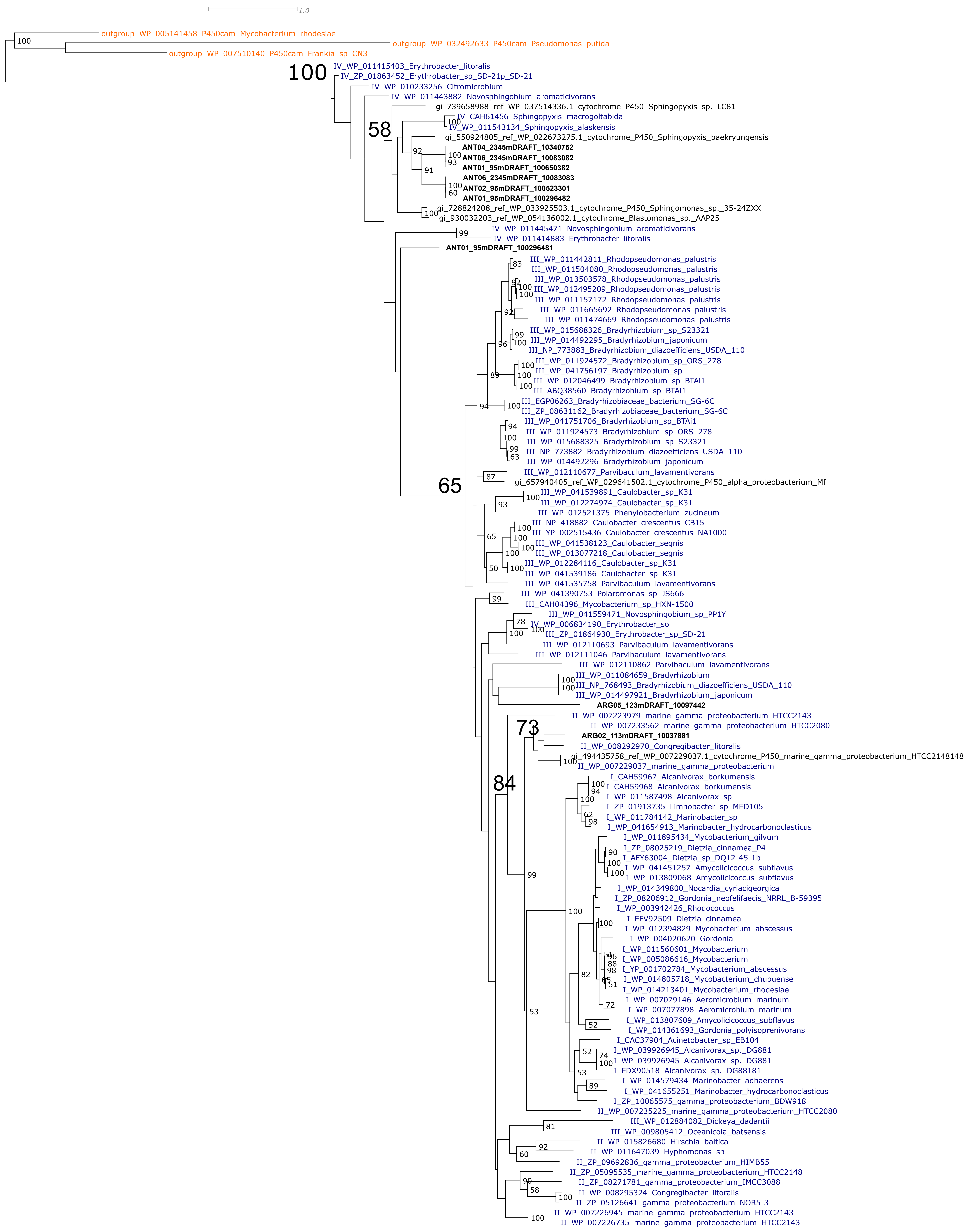

Table S3. Compound identification numbers (CID) in PubChem database of ligands (substrates and products) assayed in docking analysis.

Ligand molecule	PubChem CID
Cyclohexanone (CYH)	7967
Cyclopentanone (CYP)	8452
2-Oxo-delta(3)-4,5,5-trimethylcyclopentenylacetic acid (OTE)	441234
4-hidroxyacetophenone (4-HAP)	7469
Phenylacetone (PA)	7678
Cyclododecanona (CYD)	13246
Progesterone (PGT)	5994
Ethinoamide (Eth)	2761171
(1S,4S)-Dihydorcarvone (DHC)	443183
Bornanone (BRN)	159055
Bicyclo[3.2.0]hept-2-en-6-one (BHO)	297183
2-Phenylcyclohexanone (PCH)	95592
Androstenedione (AND)	6128
Indanone (IND)	6735
Methylphenyl sulfoxide (MPS)	14516
ε-Caprolactone	10401
3-Methyloxepan-2-one	200238
7-Methyl-2-oxepanone	543693
(4R,7R)-4 isopropenyl-7-methyloxepan-2-one	443168
(3R,6R)-6-isopropenyl-3-methyloxepan-2-one	25201860
10-methyloxecan-2-one	566646
3-methyloxecan-2-one	91147742

References

1. Huijbers, M.M.; Montersino, S.; Westphal, A.H.; Tischler, D.; van Berkel, W.J. Flavin dependent monooxygenases. *Archives of biochemistry and biophysics* **2014**, *544*, 2-17.
2. Mascotti, M.L.; Lapadula, W.J.; Ayub, M.J. The origin and evolution of baeyer—villiger monooxygenases (bvmos): An ancestral family of flavin monooxygenases. *PloS one* **2015**, *10*, e0132689.
3. Söding, J.; Biegert, A.; Lupas, A.N. The hhpred interactive server for protein homology detection and structure prediction. *Nucleic acids research* **2005**, *33*, W244-W248.
